# Multispecies Identification of Oilseed- and Meat-Specific Proteins and Heat-Stable Peptide Markers in Food Products

**DOI:** 10.3390/molecules26061577

**Published:** 2021-03-12

**Authors:** Klaudia Kotecka-Majchrzak, Natalia Kasałka-Czarna, Agata Sumara, Emilia Fornal, Magdalena Montowska

**Affiliations:** 1 Department of Meat Technology, Poznan University of Life Sciences, Wojska Polskiego 31, 60-624 Poznan, Poland; klaudia.kotecka@up.poznan.pl (K.K.-M.); natalia.czarna@up.poznan.pl (N.K.-C.); 2 Department of Pathophysiology, Medical University of Lublin, Jaczewskiego 8b, 20-090 Lublin, Poland; agatab9419@gmail.com (A.S.); emilia.fornal@umlub.pl (E.F.)

**Keywords:** food authenticity, hemp, sunflower, oilseeds, guinea fowl, rabbit meat, peptide markers, mass spectrometry

## Abstract

Consumer demand for both plant products and meat products enriched with plant raw materials is constantly increasing. Therefore, new versatile and reliable methods are needed to find and combat fraudulent practices in processed foods. The objective of this study was to identify oilseed species-specific peptide markers and meat-specific markers that were resistant to processing, for multispecies authentication of different meat and vegan food products using the proteomic LC-MS/MS method. To assess the limit of detection (LOD) for hemp proteins, cooked meatballs consisting of three meat species and hemp cake at a final concentration of up to 7.4% were examined. Hemp addition at a low concentration of below 1% was detected. The LOD for edestin subunits and albumin was 0.9% (*w*/*w*), whereas for 7S vicilin-like protein it was 4.2% (*w*/*w*). Specific heat-stable peptides unique to hemp seeds, flaxseed, nigella, pumpkin, sesame, and sunflower seeds, as well as guinea fowl, rabbit, pork, and chicken meat, were detected in different meat and vegan foods. Most of the oilseed-specific peptides were identified as processing-resistant markers belonging to 11S globulin subunits, namely conlinin, edestin, helianthinin, pumpkin vicilin-like or late embryogenesis proteins, and sesame legumin-like as well as 2S albumins and oleosin isoforms or selected enzymic proteins.

## 1. Introduction

Consumers in the 21st century have become more demanding and diverse in terms of nutrition. When meat consumption is taken into consideration, on the one hand, the number of people switching to a vegetarian diet with a range of varieties is increasing. On the other hand, people who consume animal products are more likely to reach for products with enhanced pro-health properties. Accordingly, the market for food products is constantly expanding.

Meat products are enriched with plant-derived additives, such as oils, seeds, legumes, plant extracts and protein isolates, in order to enhance the nutritional value of the product by increasing the content of dietary fibre, vitamins, phytosterols, polyphenols, and minerals, as well as to partially replace the meat-fat fraction with a vegetable-protein-fat fraction to reduce the content of saturated fatty acids and cholesterol [1,2,3,4,5]. The result is a product enriched with plant sterols and stanols that have a significant impact on human health, i.e., by lowering the concentration of cholesterol in the blood serum, especially its atherogenic low density lipoprotein fraction.

Plant products undergo many modifications to obtain analogues of meat products, e.g., vegan sausages, cutlets, burgers, pâtés, etc. For this purpose, among others, textured soy protein; fungi, including *Pleurotus sapidus* mycelium; gluten wheat; and, more recently, pea, chickpea, and protein isolates have been shown as promising substitutes for animal protein [6,7,8]. The array of available food products is wide, but what is most important is always the guarantee of health and safety. These two features are directly related to whether the composition of the food product declared by the manufacturer is authentic, i.e., whether it contains unwanted ingredients or cheaper substitutes.

The problem of food adulteration is common and has been described recently in many publications [9,10,11,12]. It is not uncommon that the composition of a product does not correspond to what is on the label and thus to the consumer’s expectations. For instance, in Operation Opson, which was conducted jointly by Europol and Interpol from December 2018 to April 2019, over €100 million worth of counterfeited and substandard food and beverages were seized [13]. Therefore, regular control of food composition is of utmost importance. Hence, there is still a need to develop new, reliable, and sensitive methods capable of simultaneous detection and identification of various ingredients of animal and plant origin in processed food as well as differentiating between their intentional and accidental presence to maintain quality standards and fulfil legal requirements in the food industry [14,15].

Many specialised analytical methods have been developed to authenticate food products, and the analytical progress in this area has been recently summarised in several articles that focused on the application of non-targeted and targeted methods or non-destructive or destructive methods, including spectroscopic techniques and omic approaches based on metabolite, protein, and DNA analyses, in some cases also in conjunction with chemometrics [11,16,17,18,19,20,21]. Among them, proteomic methods are important in authenticity investigations due to both multispecies and intraspecies detection of components derived from the same species. Based on specific proteins and peptide markers, it is possible to confidently verify the ingredients declared by the manufacturer on the label, identify allergenic proteins, and successfully demonstrate even minor irregularities in the product, especially in the case of highly processed foods.

Recently, several mass-spectrometry-based methods for simultaneous detection of meat species and/or vegetable ingredients have been introduced, e.g., eight species, pork, beef, lamb, chicken, duck, soy, peanut, and pea have been monitored in meat products based on species-specific peptide markers [22]; a qualitative LC-MS triple quadrupole multiple reaction monitoring (MRM) method has been developed to monitor duck, goose, and chicken meat simultaneously with beef and pork in highly processed meat products [23]; methods for identification of blood products made of pig, bovine, sheep, chicken, and duck blood using peptide markers [24] and for the simultaneous detection of lupine, pea, and soy proteins in meat products have been reported [25]; and, finally, peptide markers for the simultaneous detection of chicken, duck, goose, guinea fowl, ostrich, pheasant, pigeon, quail, and turkey have been tested in raw and heated meat [26]. Additionally, targeted liquid chromatography–tandem mass spectrometry (LC-MS/MS) methods were developed for multiplex detection of 14 main allergens in various food products and the simultaneous presence of milk, egg, crustaceans, and soy in fish and pork products [27,28]. The importance of reliable analytical methodologies is unquestionable because only through properly conducted analyses can it be determined whether a given food is authentic, safe, and meets consumers’ expectations, regardless of dietary preferences.

In our previous articles, we reported an absolute quantification strategy for the simultaneous detection and quantification of meat (chicken, duck, goose, pork, and beef) and allergenic protein additives (soy, milk, and egg white preparations) in meat products [29], as well as an LC-Q-TOF-MS/MS approach to differentiate rabbit and guinea fowl meat from other species in meat products based on specific peptide markers [30,31]. We also identified a set of oilseed-specific peptides in ten oilseed cakes (by-products of cold pressing oil from coconut, evening primrose, hemp, flax, milk thistle, nigella, pumpkin, rapeseed, sesame, and sunflower seeds) and further in cold-pressed oils [32,33]. The present study aimed to select thermally resistant proteins and peptide markers unique to selected oilseeds, including hemp, pumpkin, and sunflower seeds, for multispecies detection of plant and meat components in processed foods for authentication purposes, using a proteomic LC-MS/MS method carried out with a Q-TOF instrument.

## 2. Results

### 2.1. Identification of the Addition of Hemp Cake to Meatballs

In the meat industry, whole or ground oilseeds are used mostly to increase the nutritional value of meat products. We prepared meatballs containing hemp cake (HC), a by-product of cold pressing of oil from hemp seeds, which is rich in protein, fibre, and minerals, to obtain new and less-processed products that may be perceived by consumers as healthier and more environmentally friendly. HC was added in the range of 1–9.5% to estimate the limits of detection (LODs) of hemp seed proteins for authentication purposes. From a pragmatic perspective, amounts of non-declared ingredients above 1% can be associated with fraudulent practices, whereas amounts below 1% may indicate unintentional contamination. In our study, the meat was replaced HC, which gave a final concentration of the hemp additive in the cooked meatballs of 0.9%, 2.6%, 4.2%, and 7.4%, respectively (Table 1). Meatballs without the addition of HC and HC powder were used as control samples (M0, HC).

**Figure 1:**
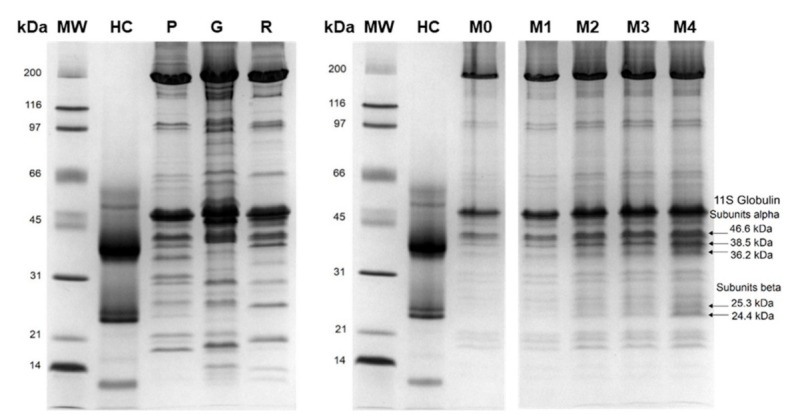
Comparison of protein profiles extracted from hemp cake, raw meat samples, and cooked meatball variants. Arrows show bands derived from hemp storage protein alpha and beta subunits detected in cooked meatballs. Lanes: HC—hemp cake; P—pork; G—guinea fowl meat; R—rabbit meat; M0—control meatball without hemp cake addition; M1, M2, M3, M4—meatballs with increasing content of hemp cake, i.e., 0.9%, 2.6%, 4.2% and 7.4%, respectively.

The complexity of the food matrix and processing operations usually makes reliable identification of various protein components difficult. Comparison of protein profiles extracted from HC, raw meat, and cooked meatballs is shown in Figure 1. Regarding hemp seeds, storage globulins are the most abundant proteins; under reducing conditions, 11S globulin monomers brake down into α and β subunits, hence an increase in the intensity of hemp-specific protein bands was observed as the content of HC increased. Similar electrophoretic profiles have been obtained previously for hemp seed meal and hemp protein isolate [34,35,36]. The experimental molecular weight for hemp globulin-specific α subunits was 38.5 and 36.2 kDa, and for β subunits it was 25.3 and 24.4 kDa (Figure 1). When protein tryptic digests were analysed using LC-MS/MS and the obtained mass spectra were compared with reference sequences gathered in the National Center for Biotechnology Information (NCBI) protein database, proteins and peptides specific to Cannabis sativa were detected with high confidence scores.

**Figure 2:**
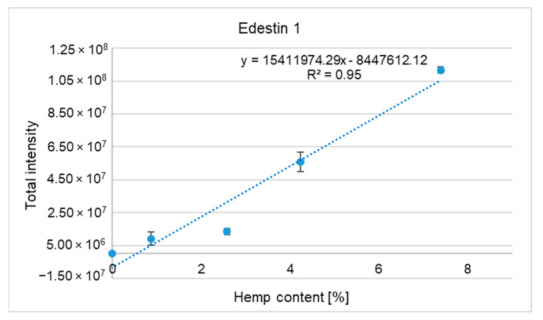
Trend line obtained for the hemp-seed-specific protein edestin 1 (CDP79023.1) from protein digests of meatballs containing 0%, 0.9%, 2.6%, 4.2%, and 7.4% hemp cake, respectively.

Table 2 presents the output scores for the five most abundant hemp proteins identified, namely edestin subunits 1–3, albumin, and 7S vicilin-like protein. The percentage of protein sequence coverage and total protein intensity increased with increasing hemp supplementation in meatballs; the highest output scores were obtained for edestin 1 (Table 2). For this protein, sequence coverage ranged from 65.9% to 78.4%. Figure 2 illustrates a trend line for edestin 1; the coefficient of determination R2 was 0.95.

Hemp-unique peptide markers detected in meatballs and HC variants are shown in Table 3. The presented peptides turned out to be resistant to thermal processing. We identified the hemp-specific peptide markers as in our preceding research on the proteomic specificity of oilseed cakes and cold-pressed oils extracted from ten seed species; however, those samples were not subjected to cooking [32,33]. In the present study, to assess the percentage LOD of hemp proteins, we examined cooked meatballs consisting of three meat species, additional ingredients, and HC at a final concentration up to 7.4%. We were able to detect the addition of hemp at low concentrations of below 1%. The LOD for edestin subunits and albumin was 0.9% (*w*/*w*), whereas for 7S vicilin-like protein it was 4.2% (*w*/*w*). Several sequences of unique peptides were detected in all meatball variants, e.g., the peptides VQVVNHMGQK and GFSVNLIQEAFNVDSETAR originating from edestin 1 and edestin 3, respectively (Table 3). This proves that the method has high discriminating power for authentication purposes but identification of the ingredients should be based on the most abundant proteins and heat-stable peptides of the tested raw material, i.e., edestin subunits in the case of hemp seeds.

Edestin accounts for about 65–70% of all storage globulins, while the albumin fraction accounts for about 25% of the storage fraction [35,36,37]. Kim and Lee [34] isolated edestin from dehulled hemp seeds of the Cheungsam variety and demonstrated its hexameric form and free radical scavenging activity. Bioactive peptides with antioxidant and antihypertensive properties have been identified from hemp seed protein hydrolysates [38,39]. These confirm the great potential of the application of hemp raw materials rich in protein in the production of functional foods as well as in the pharmaceutical field. To date, hemp proteins and mass-spectrometry-based methods have not been implemented to study the authentication of complex hemp-containing food products. Previously, LC-MS/MS methods have been developed to detect legume lupine, pea, and soy proteins simultaneously in emulsion-type sausages [25] and garden pea, meat, and honey in processed vegan or vegetarian food [40]. The established LODs for soy and lupine isolates or flours were 4 and 2 mg/kg of sausage sample, respectively [25].

### 2.2. Identification of Meat-Specific Peptides

**Figure 3:**
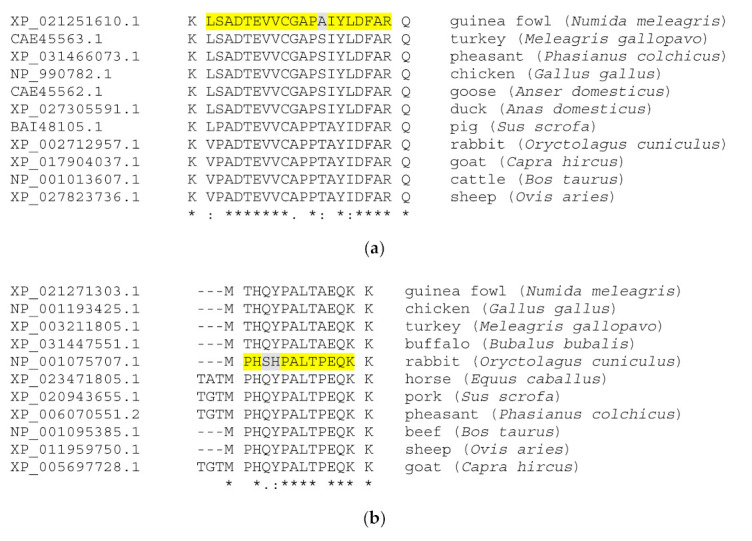
Clustal Omega protein multiple sequence alignment of guinea fowl- and rabbit-specific peptides: (**a**) triosephosphate isomerase (*Numida meleagris*) sequence fragment and (**b**) fructose-bisphosphate aldolase A (*Oryctolagus cuniculus*) sequence fragment. Identified species-specific heat-stable peptide markers are highlighted. Symbols in a multiple sequence alignment: an * (asterisk) indicates positions which have a single, fully conserved residue; a: (colon) indicates conservation between groups of strongly similar properties; a. (period) indicates conservation between groups of weakly similar properties.

The acquired mass spectra were evaluated for the simultaneous detection of hemp- and meat-specific proteins and peptides in processed products. Meatballs were made of guinea fowl meat, rabbit meat, and pork to differentiate between three types of cooked meat simultaneously, next to HC addition. Figure 1 presents the profiles of proteins extracted from the three meat species and HC, and it demonstrates the species specificity of the particular separated protein bands. As a result of the LC-MS/MS experiment, it was possible to identify over 200 proteins in the products’ digests with high confidence scores, among them muscle myofibrillar and sarcoplasmic proteins unique to guinea fowl, rabbit and pig species. Table 4 presents the Spectrum Mill output scores obtained for meat-specific proteins and species-specific peptides detected in sample M4, which contained the highest content of HC, and also chicken-specific proteins and peptides that were detected in commercial pâté with milk thistle (sample P12). The unique score is dependent on the protein abundance in the sample but also on the size of a given protein, and hence there are differences observed between individual proteins. The same meat proteins and peptides were identified in our previous studies on meat and conventional meat products, e.g., a triosephosphate isomerase peptide sequence LSADTEVVCGAPAIYLDFAR specific to guinea fowl, fructose-bisphosphate aldolase A peptide PHSHPALTPEQK specific to rabbit, pig myosin TLAFLFSGAQTGEAEAGGTK, and myosin binding protein C peptide LDVPISGEPAPTVTWK unique to chicken [29,30,31,41]. The Clustal Omega protein multiple sequence alignment of corresponding peptides obtained from triosephosphate isomerase and fructose-bisphosphate aldolase A for commonly farmed animals is shown in Figure 3. The alignment presents the uniqueness of the guinea fowl- and rabbit-specific peptides that were obtained from in silico digests. The present study confirmed the peptides’ specificity for authenticating complex food matrices consisting of animal and plant raw materials.

It is worth emphasising that we kept the analytical procedure as simple as possible to avoid compromising the analytical throughput of the method. The implemented proteomic approach is robust and reliable and thus competitive with other LC-MS-based methods. The sample was homogenised in a mild buffer, digested in-solution, and then LC-Q-TOF-MS/MS was applied to the purified digest. Previously, using various mass-spectrometry-based techniques, heat-stable peptide markers have been identified in cooked meats of five species, i.e., sheep, cattle, pig, duck, and chicken [42]. The same five meat species next to peanut powder, pea powder, and soybean meal have been screened for multi-species-specific peptides in heat-treated samples, and the LOD for any of the eight species in three- or four-component mixtures was set at 0.5% contamination [22]. An LC-MS method has been developed to identify and quantify eight meat species (duck, rabbit, chicken, turkey, buffalo, equine, deer, and sheep) specifically in Bolognese sauce based on eight species-specific peptide markers [43]. LODs for a particular species calculated in the sauce were in the range of 0.2–0.8%. Peptides derived from haemoglobin, apolipoprotein A-I, and carbonic anhydrase have been identified as markers for animal blood products of different animal species, i.e., pig, bovine, sheep, chicken, and duck [24]. And finally, the proteomic approach has been evaluated for the simultaneous detection of nine poultry species in raw and heated meat [26].

### 2.3. Identification of Unique Proteins and Peptides of Oilseeds in Commercial Food Products

In the next stage, we applied our previously established MS-based methodology for detection and identification of oilseed peptide markers in various commercially processed products. The aim was to select peptides potentially resistant to the heat and technological processes applied in the food industry. A set of species-specific peptide markers unique to coconut kernel, flaxseed, hemp seeds, milk thistle, nigella, pumpkin seeds, rapeseed, sesame and sunflower seeds was evaluated. All these peptides were identified in HC and cold-pressed oils of the given species [32,33]. In this work, a proteomic analysis was carried out on 12 ready-to-eat food products of different compositions (Table 1), containing oilseeds and/or vegetable oils. A list of all oilseed peptide markers identified in this study for the different food products is presented in Table S1 (Supplementary Materials). Most of the detected peptides were identified as heat-stable markers belonging to 11S globulin subunits, namely flax conlinin, the aforementioned edestin, helianthinin, pumpkin vicilin-like or late embryogenesis proteins, and sesame legumin-like as well as 2S albumins and oleosin isoforms or selected enzymic proteins.

Sunflower seeds turned out to be a very popular ingredient for vegan foods; the seeds were present and specific proteins and peptides were identified in six out of twelve examined products. Peptides derived from three proteins were observed in all six samples, i.e., four unique peptides of 2S seed storage albumin 2 precursor, GQFGGQEMDIAR, AQILPNVCNLQSR, SQQCSETEIQRPVSQCQR, ECQCEAVQEVAR; a putative 11-S seed storage peptide SPFGGQEELTR; and the 2S seed storage albumin 1 peptide GQFGGQEMETAR (Table S1, Supplementary Materials). Additionally, considerable amounts of peptides unique to hemp seed and pumpkin seed were identified in processed products. Bio vegan pumpkin pâté (sample P1) should contain 49% pumpkin seeds according to the information on the package, whereas hemp pâté (sample P8) should consist of hemp seeds (9%), sunflower seeds, and pumpkin seeds (quantity not declared); nevertheless, the identification of specific pumpkin peptides in both products was at a similar level. Hence, the selected unique proteins and peptides are good markers for testing the authenticity of products containing pumpkin seeds; similar can be claimed for the identified hemp and sunflower peptide markers.

When coconut fat/oil was declared in vegan foods, characteristic coconut proteins and peptides were not detected, even though, for instance, the manufacturer declared 21% coconut oil content in the chicken-flavoured sausage (sample P11). Similarly, none of the rapeseed-specific proteins and peptides were identified in five types of pâtés containing rapeseed oil (Supplementary Materials, Table S1). The likely reason could be the manufacturer’s use of refined vegetable oils. In sample P12, which was chicken pâté with milk thistle, neither of the two preprosilpepsin 2 peptides unique to milk thistle were found. A milk thistle content of 8% was declared on the label, and its addition was distinctly visible, while the lack of proteomic data obtained for milk thistle may indicate that the protein and peptides were susceptible to heating and degraded during industrial processing. On the other hand, of two specific nigella proteins, nigellin and thionin, three nigellin heat-stable peptides were detected in vegan pâté (sample P7). This indicates both good sensitivity and specificity of the method and that the peptides may be good markers for processed products, as the total declared nigella content in the product was low (1%).

Sesame was present in mini burgers (sample P4), and several identical unique peptides originating from sesame 11S globulins, 7S globulin, and 2S albumin were found either in the sesame cake or cold-pressed sesame oil in our preceding studies [32,33]. Moreover, the presence of one of the seven sesame allergens, namely Ses i 3 (7S vicilin-like globulin), has been confirmed due to the identification of its specific peptide IPYVFEDQHFITGFR. Two pumpkin-specific peptides, NVDEECRCDMLEEIAR and NLPSMCGIRPQR, derived from pumpkin allergenic 2S albumin (Cuc ma 5), have been identified in the present work. Six out of seven sesame allergens and the pumpkin allergen Cuc ma 5 have been previously detected in cold-pressed oil [32]. Recently, a targeted proteomic approach has been developed for the detection and quantification of the seven sesame allergens in raw seeds and different products including sauces, cookies, cake, and candy [44]. LODs for the sesame marker peptides were determined to range from 0.1 to 140.0 fmol/μL.

To date, research articles on multispecies identification of oilseed- and meat-specific proteins and heat-stable peptide markers in various food products are scarce. The current study presents a set of unique processing-resistant peptide markers and confirms their utility in the authentication of the plant or meat components/additives used in different commercially manufactured products. However, international protein databases are not complete in the area of oilseed protein sequences and other plant matrices. Since information on oilseed proteins accounts for approximately a few percent of entries in protein databases, this provides an excellent space for further proteomics research.

## 3. Materials and Methods

### 3.1. Reagents and Samples

Acetonitrile (LC-MS grade) and formic acid (MS grade) were obtained from Sigma-Aldrich (Schnelldorf, Germany). Sequence-grade modified trypsin gold, lyophilized, was obtained from Promega GmbH (Mannheim, Germany). Reversed-phase Sep-Pak C18 Plus cartridges, sorbent weight 360 mg/0.7 mL, were obtained from Waters (Milford, MA, USA). All other chemicals were purchased from Sigma-Aldrich at the best available purity grade.

The samples for the study (*n* = 18) consisted of five variants of pork meatballs differing in the quantity of hemp cake, as well as food products purchased at stationary and on-line stores. The commercial products contained various oilseeds. Table 1 presents the meat and oilseed species composition of the analysed samples. The samples were analysed in triplicate. Proteins and peptides derived from guinea fowl (*Numida meleagris*), rabbit (*Oryctolagus cuniculus*), pig (*Sus scrofa*), chicken (*Gallus gallus*), coconut (*Cocos nucifera* L.), hemp (*Cannabis sativa* L.), flax (*Linum usitatissimum* L.), milk thistle (*Silybum marianum* L.), nigella/black cumin (*Nigella sativa* or *N. indica*), pumpkin (*Cucurbita pepo* L.), rapeseed (*Brassica napus* L.), sesame (*Sesamum indicum* L.), and sunflower (*Helianthus annuus* L.) were examined in the present study.

### 3.2. Preparation of Meatballs

Hemp seeds (*Cannabis sativa* L.) of the Finola variety were obtained from the Polish company SemCo Sp. z o.o. (Szamotuły near Poznań, Poland). The hemp cake was prepared by the cold pressing process using a Yoda oil press YD-ZY-02A (Warsaw, Poland); the remaining cake was dried overnight in a drying oven at 40 °C and then ground in a grinder (Bosch GmbH, Gerlingen-Schillerhöhe, Germany). The pork meat was purchased at a local store (Poznań, Poland); rabbit and guinea fowl meats were purchased at Makro Cash & Carry hypermarket (Poznań, Poland). The meat was stripped of bones and skin and then ground using an electric Zelmer type 685.5 mincer (Zelmer S.A., Rzeszów, Poland) through a mesh size of 3 mm. Subsequently, the minced meat was divided into five equal portions and mixed with water, breadcrumbs, salt and spices, and finally, 0%, 1%, 3.1%, 5.2%, and 9.5% hemp cake was added into each portion based on total meat weight percentage. The batter was mixed manually until the ingredients were spread evenly. The percentage of hemp additive in the final meatballs was in the range of 0.9% to 7.4% (Table 1). The batter was left for approximately 30 min before the meatballs (50 ± 1 g) were formed. Each variant was placed on a stainless-steel baking tray and heated in a Rational Combi convection oven model SCC 61 (Landsberg am Lech, Germany). Heating was carried out at a temperature of 160 °C, with an air humidity in the oven chamber of 75%, until reaching a core temperature of 72 °C. The meatballs were vacuum-packed in barrier bags using a Multivac C100 chamber machine and stored at −80 °C until further protein analysis.

### 3.3. Sodium Dodecyl Sulphate–Polyacrylamide Gel Electrophoresis (SDS-PAGE)

SDS-PAGE was performed to compare the profiles of the proteins extracted from the hemp cake, meat species and meatball variants. Samples were homogenised in 0.1 mol/L of aqueous ammonium bicarbonate, using a T25 Ultra-Turrax (IKA Labortechnik, Staufen, Germany) at 9500 rpm for 2 × 20 s, followed by 13,500 rpm for 30 s, and then vacuum-dried using a miVacDuo Concentrator (Genevac Ltd., Suffolk, UK). Dried samples (5 mg) were dissolved with lysis buffer (8 M urea, 2 M thiourea, 0.05 mM Tris, 75 mM dithiothreitol (DTT), 3% SDS, and 0.05% bromophenol blue, at pH = 6.8) and heated at 98 °C for 4 min. Protein concentration was determined using a 2-D Quant kit (GE Healthcare Bio-Sciences, Fairfield, CT, USA). Protein aliquots (12 μg) were loaded onto 15% polyacrylamide gels prepared in a Hoefer SE250 system (GE Healthcare Bio-Sciences, version). A reference broad-range molecular weight standard (Bio-Rad Laboratories, Inc., Hercules, CA, USA) was applied. Gels were run at a constant current of 20 mA per gel, then stained with Coomassie brilliant blue and scanned (Gel Doc XR+ System, Bio-Rad Laboratories, Inc., CA, USA) and processed using Image Lab 6.0.1 software (Bio-Rad Laboratories).

### 3.4. In-Solution Tryptic Digestion

Protein digestion was carried out as previously described [45]. Dried samples (5 mg) were rehydrated in 100 μL of 50 mM ammonium bicarbonate. The proteins were reduced by 200 mM DTT (56 °C for 1 h) and then alkylated using 200 mM iodoacetamide for 30 min in the dark at room temperature. The remaining iodoacetamide was quenched by the addition of 200 mM DTT and incubated at room temperature for 30 min. The samples were digested in an ammonium bicarbonate solution containing 0.083 μg/μL of trypsin (Promega GmbH, Mannheim, Germany) at 37 °C overnight. The digests were purified by reversed-phase extraction using Sep-Pak C18 Plus cartridges (Waters, Milford, MA, USA). The solid-phase extraction (SPE) column was equilibrated with solvent A consisting of 98% water, 2% acetonitrile, and 0.1% formic acid, then with solvent B consisting of 65% acetonitrile, 35% water, and 0.1% formic acid. The sample (0.6 mL) was then added to the cartridge and washed with solvent A. The peptides were eluted with solvent B and vacuum-dried in a centrifugal evaporator (miVacDuo Concentrator, Genevac Ltd., Suffolk, UK). Samples were resuspended in 2% acetonitrile, in Milli-Q water containing 0.1% formic acid (solvent A), before UHPLC-Q-TOF-MS/MS analyses.

### 3.5. Protein and Peptide Identification

Mass spectrometry analysis was performed according to a previously described procedure [32] on an Agilent Technologies 1290 Infinity series liquid chromatograph (Santa Clara, CA, USA), composed of a binary pump and an autosampler coupled to a 6550 UHD iFunnel Q-TOF LC/MS (Agilent Technologies, Santa Clara, CA, USA). Compounds were ionised by electrospray ionisation (ESI) using a Jet Stream Technology ion source. Chromatographic separation was performed on a 2.1 × 150 mm, 1.8 μm particle-size Agilent Rapid Resolution High Definition (RRHD) Eclipse Plus C18 column. Instrument control and data acquisition were performed using Agilent MassHunter Workstation B.09 software. The LC parameters were set as follows: 10 μL injection volume and 0.3 mL/min mobile phase flow. The mobile phase consisted of 0.1% formic acid in water (solvent A) and 0.1% formic acid in acetonitrile (solvent B). Gradient steps were applied as follows: 0–2 min, 2% B; 2–40 min, to 32% B; 40–45 min, to 37% B; 45–50 min, to 90% B; 50–55 min, 90% B; and a 5-min post-run at 2% B. The ion source gas (nitrogen) temperature was 250 °C, the flow rate was 14 L/min, the nebulizer pressure was 35 psi, the sheath gas temperature was 250 °C, and the sheath gas flow was 11 L/min. The capillary voltage was set to 3500 V, the nozzle voltage to 1000 V, and the fragmentor to 400 V. Positive ions formed in an electrospray were acquired in the range of 100–1700 *m/z* in MS scan mode and in auto MS/MS mode, with a scan rate of 5 scan/s for MS and 3 scan/s for MS/MS. Internal mass calibration was enabled using two reference masses at 121.0509 and 922.0098 *m/z*.

The National Center for Biotechnology Information (NCBI, U.S. National Library of Medicine) protein database search for protein and peptide identification was performed using Spectrum Mill MS Proteomics Workbench with >70% score peak intensity and 10 ppm precursor mass tolerance, with the following parameters: trypsin enzyme, taxonomy of a given animal or plant species or a given taxonomy genus, a maximum of two missed cleavages, 50 ppm products mass tolerance, carbamidomethylation as a fixed modification, and methionine oxidation as a variable modification. The matches and Spectrum Mill scores were evaluated at a 1% false discovery rate (FDR) for identity and homology thresholds. Selected peptides, in FASTA format, were searched against the NCBInr database, using the protein Basic Local Alignment Search Tool (BLAST) and blastp algorithm (U.S. National Library of Medicine, Bethesda, MD, USA) for species and protein specificity.

## 4. Conclusions

Consumer demand for both plant products and meat products enriched with plant raw materials is constantly increasing. Oilseeds as a source of amino acids and unsaturated fats are increasingly used to partially replace proteins and animal fatty acids in meat products, as well as to produce their vegetable analogues. This study examined the applicability of the proteomic approach to detect species-specific peptide markers in different types of processed food for authentication purposes. A set of peptide markers both unique to selected oilseed and meat species and resistant to thermal processing can be helpful to verify the declared composition of various food products. Specific peptides unique to hemp seeds, flaxseed, nigella, pumpkin, sesame, and sunflower seeds, as well as guinea fowl, rabbit, pork, and chicken meat, were detected in meat and vegan foods. The heat-stable unique oilseed peptides were released mainly from specific 11S and 2S seed storage proteins, oleosins and several enzymic proteins. The method can be implemented to identify particular protein ingredients of plant and/or animal origin simultaneously and can thus be used as an alternative to various enzymic and nucleic acid tests to study authentication issues in complex food matrices.

## Figures and Tables

**Table 1:** Meat and oilseed species composition of the analysed food products.

Sample	Product	Analysed Ingredient	Other Ingredients
M0	meatballs (control)	pork (66%), rabbit meat (10.9%), guinea fowl meat (7.3%)	water (8.9%), bread crumbs (5.3%), salt (1.3%), pepper (0.2%), garlic (0.1%)
M1	hemp meatballs	pork (65.4%), rabbit (10.8%), guinea fowl meat (7.2%), hemp cake (0.9%)	water (8.8%), bread crumbs (5.3%), salt (1.3%), pepper (0.2%), garlic (0.1%)
M2	hemp meatballs	pork (64.3%), rabbit (10.6%), guinea fowl meat (7.1%), hemp cake (2.6%)	water (8.6%), bread crumbs (5.2%), salt (1.3%), pepper (0.2%), garlic (0.1%)
M3	hemp meatballs	pork (63.2%), rabbit (10.4%), guinea fowl meat (7%), hemp cake (4.2%)	water (8.5%), bread crumbs (5.1%), salt (1.3%), pepper (0.2%), garlic (0.1%)
M4	hemp meatballs	pork (61.1%), rabbit (10.1%), guinea meat (6.7%), hemp cake 7.4%)	water (8.2%), bread crumbs (4.9%), salt (1.2%), pepper (0.2%), garlic (0.1%)
HC	hemp cake	hemp cake (100%)	-
P1	bio vegan pumpkin pâté	pumpkin seeds (49%), rapeseed oil	corn grits, buckwheat bran, onion, marjoram, garlic, nutmeg, salt
P2	four grain pâté	hulled sunflower seeds, flaxseed, coconut fat	lentils, chickpeas, potato flour, buckwheat flour, carrots, parsley, celery, leek, olive oil, soy sauce, spices, salt
P3	vegetable paste	rapeseed oil (5%), sunflower seeds	chickpeas, onion (5%), celery, water, dried tomatoes, dried candied cranberries, salt, sugar, garlic, basil, black pepper, acidity regulator: citric acid
P4	mini burgers	sunflower seeds, sesame	rice, zucchini (17%), tofu (17%), carrots (15%), spelled flakes, oatmeal, spelled flour, buckwheat flour, bean sprouts (2%), sesame, salt, spices, sunflower oil
P5	bio sunflower pâté	sunflower seeds (25%), rapeseed oil	millet, buckwheat bran, onion, marjoram, nutmeg, pepper, salt
P6	bio sandwich paste	sunflower oil (20%), sunflower seeds (14%)	tomato paste (30%), tomato puree (12%), water, lemon juice, concentrated apple juice, basil (3%), salt, potato starch
P7	vege pâté	nigella seeds (1%), rapeseed oil	chickpeas (60%), millet, carrots, salt, garlic, spices
P8	hemp pâté	hulled hemp seeds (9%), hulled sunflower seeds, rapeseed oil, pumpkin seeds	onion, garlic, yeast, salt, natural spices, glucose, guar gum
P9	bio hemp spacebar	coconut fat, hemp seeds (2%)	seitan (86%), salt, yeast extract, spices, onion, locust bean gum, guar gum, natural beech wood smoke
P10	vegetarian balls	hemp seeds (5%), sunflower oil	wheat (43%), carrots, sweet potatoes (11%), cornflour, red pepper, onion, Emmentaler (6%), arugula (1%), salt, spices
P11	chicken flavored sausage	coconut oil (21%)	water, modified starch, barley starch, rice protein, salt, apple juice concentrate, chicken flavor, citric acid, olive extract, vegetable and fruit concentrate (pepper, carrot, radish, apple, black currant), vitamin B12
P12	pâté with milk thistle	chicken (37%), milk thistle (8%)	celery, chicken liver, eggs, carrots, onion, parsley, salt, bread crumbs, spices

**Table 2:** Hemp-seed-specific proteins identified in hemp cake and cooked meatballs using LC-QTOF-MS/MS.

Protein (Accession No.)	Sample	Sequence Coverage (%)	Matched Peptides	Unique Score	Total Intensity (*n* = 3)
edestin 1 (CDP79023.1)	M1	65.9	28	411.49	4.08 × 10^7^
	M2	65.7	27	415.23	6.11 × 10^7^
	M3	69.8	32	551.19	2.94 × 108
	M4	74.9	37	627.60	4.29 × 10^8^
	HC	78.4	45	787.00	2.43 × 10^9^
edestin 2 (CDP79028.1)	M1	57.4	23	344.04	4.70 × 10^7^
	M2	57.4	23	359.00	7.42 × 10^7^
	M3	68.8	28	503.93	3.77 × 10^8^
	M4	72.9	36	637.90	3.98 × 10^8^
	HC	66.8	42	775.24	1.89 × 10^9^
edestin 3 (SNQ45160.1)	M1	59.4	23	321.57	2.84 × 10^7^
	M2	48.4	21	320.6	3.74 × 10^7^
	M3	69.4	27	457.01	2.45 × 10^8^
	M4	72.3	31	517.93	6.24 × 10^8^
	HC	82.6	41	731.50	2.99 × 109
albumin (SNQ45151.1)	M1	19.0	3	32.14	1.17 × 10^6^
	M2	23.9	4	52.36	4.90 × 10^6^
	M3	34.5	6	78.89	1.70 × 10^7^
	M4	34.5	6	90.47	3.84 × 10^7^
	HC	41.5	9	142.84	1.53 × 108
7S vicilin-like protein (SNQ45153.2)	M3	7.5	3	34.37	5.66 × 10^5^
	M4	25.7	10	138.97	2.19 × 10^6^
	HC	45.6	23	386.04	6.87 × 10^7^

**Table 3:** Hemp-seed-specific peptides detected in hemp cake and cooked meatballs.

Protein	Peptide Sequence	M1	M2	M3	M4	HC
edestin 1 (CDP79023.1)	NAIYTPHWNVNAHSVMYVLR	+	+	+	+	+
	YLEEAFNVDSETVK	+	+	+	+	+
	YTIQQNGLHLPSYTNTPQLVYIVK	+	+	+	+	+
	ISTVNSYNLPILR	+	+	+	+	+
	VEAEAGLIESWNPNHNQFQCAGVAVVR			+	+	+
	GILGVTFPGCPETFEESQR		+	+	+	+
	GQGQGQSQGSQPDR			+	+	+
	QASSDGFEWVSFK	+	+	+	+	+
	VQVVNHMGQK	+	+	+	+	+
	EETVLLTSSTSSR		+	+	+	+
	LQGQNDDR				+	+
	GTLDLVSPLR		+	+	+	+
	QQNQCQIDR		+	+	+	+
edestin 2 (CDP79028.1)	ILAESFNVDTELAHK	+	+	+	+	+
	AMPDDVLANAFQISR	+	+	+	+	+
	NGMMAPHFNLDSHSVIYVTR	+	+	+	+	+
	GLLLPSFLNAPMMFYVIQGR	+	+	+	+	+
	ASAQGFEWIAVK			+	+	+
	SEGASSDEQHQK				+	+
	LNTLNNYNLPILR	+	+	+	+	+
	DEISVFSPSSQQTR		+		+	
	WQSQCQFQR			+	+	+
	LQVVDDNGR	+	+	+	+	+
	GEDLQIIAPSR	+		+	+	+
edestin 3 (SNQ45160.1)	VECEGGMIESWNPNHEQFQCAGVALLR				+	+
	FYIAGNPHEDFPQSR	+	+	+	+	+
	AMPEDVIANSYQISR		+	+	+	+
	GFSVNLIQEAFNVDSETAR	+	+	+	+	+
	LTIQPNGLHLPSYTNGPQLIHVIR	+	+	+	+	+
	TAVYGDQNECQLNR			+	+	+
	GVLGTLFPGCAETFEEAQVSVGGGR			+	+	+
	NAMYAPHYNINAHSIIYAIR				+	+
	LEACEPDHR					+
	QGQALTVPQNFAVVK	+	+	+	+	+
	FYIAGNPHQEFPQSMMTQQGR					+
albumin (SNQ45151.1)	CPALEMEIQK			+	+	+
	NIPSMCGMQPR	+	+	+	+	+
7S vicilin-like protein (SNQ45153.2)	GPELAAAFGLSLER			+	+	+
	EILSSQQEGPIVYIPDSR					+
	NNYGWSIALDEFSYSPLR					+

**Table 4:** Guinea fowl, rabbit, pig, and chicken meat-specific proteins and peptides detected in food products (samples M4 and P12).

Protein	Accession No.	Sequence Coverage (%)	Matched Peptides	Unique Score	Total Intensity (n = 3)	Peptide Sequence
Guinea fowl (*Numida meleagris*)						
triosephosphate isomerase	XP_021251610.1	78.6	16	251.86	2.12 × 10^7^	LSADTEVVCGAPAIYLDFAR
phosphoglycerate mutase 1	XP_021255837.1	47.2	9	131.51	1.82 × 10^7^	HLESMSEEAIMELNLPTGIPIVYELDK
Rabbit (*Oryctolagus cuniculus*)						
myosin heavy chain	NP_001103286.1	68.1	186	3179.44	1.71 × 10^9^	TLAFLFTGTAAAEAEGGGK
myosin heavy chain, skeletal muscle	XP_008268944.1	72.5	180	2981.90		TLAFLFSGAQTGEEGGGGGK
fructose-bisphosphate aldolase A	NP_001075707.1	78.7	27	421.31	1.41 × 10^9^	PHSHPALTPEQK
ATP-dependent 6-phosphofructokinase, muscle type	XP_002723486.1	29.1	17	250.81	1.18 × 10^7^	ALVFQPVTELQNQTDFEHR
beta globin	AAA02985.1	53.7	6	99.36	7.09 × 10^6^	FFESFGDLSSAHAVMSNPK
						VLAAFSEGLNHLDNLK
Pig (*Sus scrofa*)						
myosin-1	NP_001098421.1	74.7	205	3512.06	2.12 × 10^9^	TLAFLFTGAAGADAEAGGGK
myosin-2 isoform X1	XP_020921875.1	71.3	194	3311.78	1.76 × 10^9^	TLAFLFSGAQTGEAEAGGTK
myosin-4	NP_001116613.1	74.0	190	3190.20	1.71 × 10^9^	TLAFLFAER
myosin-7	NP_999020.2	71.4	184	3007.08	1.13 × 10^9^	LLSNLFANYAGADTPVEK
albumin, partial	CAA30970.1	72.3	43	676.67	6.21 × 10^7^	TVLGNFAAFVQK
carbonic anhydrase 3	NP_001008688.1	81.1	17	272.82	5.96 × 10^7^	HDPSLLPWTASYDPGSAK
hemoglobin (beta subunit)	pdb|1QPW|B	85.6	12	212.07	2.28 × 10^7^	FFESFGDLSNADAVMGNPK
glyceraldehyde-3-phosphate dehydrogenase	NP_001193288.1	90.0	26	445.78	3.92 × 10^8^	WGDAGATYVVESTGVFTTMEK
Chicken (*Gallus gallus*)						
myosin binding protein C	NP_001038124.1	46.5	40	617.87	3.28 × 10^7^	TSDVDSVFFIR
						LDVPISGEPAPTVTWK
						VAGAALPCAPAVK
pyruvate kinase	NP_990800.1	79.2	35	588.24	1.16 × 10^8^	EPADAMAAGAVEASFK
beta-enolase	NP_990450.1	54.8	21	387.40	1.29 × 10^8^	LAMQEFMVLPVGAASFHDAMR
sarcoplasmic/endoplasmic reticulum calcium ATPase 1	NP_990850.1	46.2	42	649.66	8.81 × 10^7^	IGIFTEDEEVSGR

## Data Availability

The datasets generated and analysed during this study are available from the corresponding author on reasonable request.

## Supplementary Materials

The following are available online. Table S1: Peptides unique to oilseed proteins analysed in commercial food products.

## References

[1] Bilek A.E., Turhan S. (2009). Enhancement of the nutritional status of beef patties by adding flaxseed flour. Meat Sci..

[2] Frassinetti S., Moccia E., Caltavuturo L., Gabriele M., Longo V., Bellani L., Giorgi G., Giorgetti L. (2018). Nutraceutical potential of hemp (*Cannabis sativa* L.) seeds and sprouts. Food Chem..

[3] Kotecka-Majchrzak K., Sumara A., Fornal E., Montowska M. (2020). Oilseed proteins—Properties and application as a food ingredient. Trends Food Sci. Technol..

[4] Novello D., Schiessel D.L., Santos E.F., Pollonio M.A.R. (2019). The effect of golden flaxseed and by-product addition in beef patties: Physicochemical properties and sensory acceptance. Int. Food Res. J..

[5] Zając M., Guzik P., Kulawik P., Tkaczewska J., Florkiewicz A., Migdał W. (2019). The quality of pork loaves with the addition of hemp seeds, de-hulled hemp seeds, hemp protein and hemp flour. LWT.

[6] Kumar P., Chatli M.K., Mehta N., Singh P., Malav O.P., Verma A.K. (2017). Meat analogues: Health promising sustainable meat substitutes. Crit. Rev. Food Sci. Nutr..

[7] Stephan A., Ahlborn J., Zajul M., Zorn H. (2017). Edible mushroom mycelia of *Pleurotus sapidus* as novel protein sources in a vegan boiled sausage analog system: Functionality and sensory tests in comparison to commercial proteins and meat sausages. Eur. Food Res. Technol..

[8] Lam A.C.Y., Karaca A.C., Tyler R.T., Nickerson M.T. (2018). Pea protein isolates: Structure, extraction, and functionality. Food Rev. Int..

[9] Ehmke M.D., Bonanno A., Boys K., Smith T.G. (2019). Food fraud: Economic insights into the dark side of incentives. Aust. J. Agric. Resour. Econ..

[10] Esteki M., Regueiro J., Simal-Gándara J. (2019). Tackling Fraudsters with Global Strategies to Expose Fraud in the Food Chain. Compr. Rev. Food Sci. Food Saf..

[11] Medina S., Perestrelo R., Silva P., Pereira J.A.M., Câmara J.S. (2019). Current trends and recent advances on food authenticity technologies and chemometric approaches. Trends Food Sci. Technol..

[12] Tibola C.S., Alves da Silva S., Dossa A.A., Patrício D.I. (2018). Economically Motivated Food Fraud and Adulteration in Brazil: Incidents and Alternatives to Minimize Occurrence. Int. J. Food Sci..

[13] Illicit Food and Drink Seized in Global Operation. https://www.interpol.int/News-and-Events/News/2019/Illicit-food-and-drink-seized-in-global-operation.

[14] Brockmeyer J. (2018). Novel approaches for the MS-based detection of food allergens: High resolution, MS3, and beyond. J. AOAC Int..

[15] Stoyke M., Becker R., Brockmeyer J., Jira W., Popping B., Uhlig S., Wittke S. (2019). German Government Official Methods Board Points the Way Forward: Launch of a New Working Group for Mass Spectrometry for Protein Analysis to Detect Food Fraud and Food Allergens. J. AOAC Int..

[16] Creydt M., Fischer M. (2018). Omics approaches for food authentication. Electrophoresis.

[17] He Y., Bai X., Xiao Q., Liu F., Zhou L., Zhang C. (2020). Detection of adulteration in food based on nondestructive analysis techniques: A review. Crit. Rev. Food Sci. Nutr..

[18] Li Y.-C., Liu S.-Y., Meng F.-B., Liu D.-Y., Zhang Y., Wang W., Zhang J.-M. (2020). Comparative review and the recent progress in detection technologies of meat product adulteration. Compr. Rev. Food Sci. Food Saf..

[19] Sajali N., Wong S.C., Abu Bakar S., Mokhtar N.F.K., Manaf Y.N., Yuswan M.H., Desa M.N.M. (2020). Analytical approaches of meat authentication in food. J. Food Sci. Technol..

[20] Valand R., Tanna S., Lawson G., Bengtström L. (2020). A review of Fourier Transform Infrared (FTIR) spectroscopy used in food adulteration and authenticity investigations. Food Addit. Contam. Part A.

[21] Stachniuk A., Sumara A., Montowska M., Fornal M. (2021). Liquid chromatography–mass spectrometry bottom-up proteomic methods in animal species analysis of processed meat for food authentication and the detection of adulterations. Mass Spectrom. Rev..

[22] Li Y., Zhang Y., Li H., Zhao W., Guo W., Wang S. (2018). Simultaneous determination of heat stable peptides for eight animal and plant species in meat products using UPLC-MS/MS method. Food Chem..

[23] Fornal E., Montowska M. (2019). Species-specific peptide-based liquid chromatography–mass spectrometry monitoring of three poultry species in processed meat products. Food Chem..

[24] Zhang Y., Wang S., Ma Y., Li H., Li Y. (2020). Identification and absolute quantification of animal blood products by peptide markers using an UPLC–MS/MS method. Eur. Food Res. Technol..

[25] Hoffmann B., Münch S., Schwägele F., Neusüß C., Jira W. (2017). A sensitive HPLC-MS/MS screening method for the simultaneous detection of lupine, pea, and soy proteins in meat products. Food Control.

[26] Häfner L., Kalkhof S., Jira W. (2021). Authentication of nine poultry species using high-performance liquid chromatography–tandem mass spectrometry. Food Control.

[27] Croote D., Braslavsky I., Quake S.R. (2019). Addressing complex matrix interference improves multiplex food allergen detection by targeted LC-MS/MS. Anal. Chem..

[28] Stella R., Sette G., Moressa A., Gallina A., Aloisi A.M., Angeletti R., Biancotto G. (2020). LC-HRMS/MS for the simultaneous determination of four allergens in fish and swine food products. Food Chem..

[29] Montowska M., Fornal E. (2019). Absolute quantification of targeted meat and allergenic protein additive peptide markers in meat products. Food Chem..

[30] Stachniuk A., Sumara A., Montowska M., Fornal M. (2020). LC-QTOF-MS identification of rabbit-specific peptides for authenticating the species composition of meat products. Food Chem..

[31] Stachniuk A., Sumara A., Montowska M., Fornal E. (2021). Peptide markers for distinguishing guinea fowl meat from that of other species using liquid chromatography-mass spectrometry. Food Chem..

[32] Kotecka-Majchrzak K., Sumara A., Fornal E., Montowska M. (2020). Identification of species-specific peptide markers in cold-pressed oils. Sci. Rep..

[33] Kotecka-Majchrzak K., Sumara A., Fornal E., Montowska M. (2021). Proteomic analysis of oilseed cake: A comparative study of species-specific proteins and peptides extracted from ten seed species. J. Sci. Food Agric..

[34] Kim J.J., Lee M.Y. (2011). Isolation and characterization of edestin from Cheungsam hempseed. J. Appl. Biol. Chem..

[35] Malomo S.A., Rong H., Aluko R.E. (2014). Structural and functional properties of hemp seed protein products. J. Food Sci..

[36] Mamone G., Picariello G., Ramondo A., Nicolai M.A., Ferranti P. (2019). Production, digestibility and allergenicity of hemp (*Cannabis sativa* L.) protein isolates. Food Res. Int..

[37] Aiello G., Fasoli E., Boschin G., Lammi C., Zanoni C., Citterio A., Arnoldi A. (2016). Proteomic characterization of hempseed (*Cannabis sativa* L.). J. Proteom..

[38] Girgih A.T., He R., Malomo S., Offengenden M., Wu J., Aluko R.E. (2014). Structural and functional characterization of hemp seed (*Cannabis sativa* L.) protein-derived antioxidant and antihypertensive peptides. J. Funct. Foods.

[39] Orio L.P., Boschin G., Recca T., Morelli C.F., Ragona L., Francescato P., Arnoldi A., Speranza G. (2017). New ACE-inhibitory peptides from hemp seed (*Cannabis sativa* L.) proteins. J. Agric. Food Chem..

[40] Huschek G., Bönick J., Merkel D., Huschek D., Rewel H. (2018). Authentication of leguminous-based products by targeted biomarkers using high resolution time of flight mass spectrometry. LWT Food Sci. Technol..

[41] Montowska M., Alexander M.R., Tucker G.A., Barrett D.A. (2014). Rapid detection of peptide markers for authentication purposes in raw and cooked meat using ambient Liquid Extraction Surface Analysis Mass Spectrometry. Anal. Chem..

[42] Wang G.-J., Zhou G.-Y., Ren H.-W., Xu Y., Yang Y., Guo L.-H., Liu N. (2018). Peptide biomarkers identified by LC–MS in processed meats of five animal species. J. Food Compos. Anal..

[43] Prandi B., Varani M., Faccini A., Lambertini F., Suman M., Leporati A., Tedeschi T., Sforza S. (2019). Species specific marker peptides for meat authenticity assessment: A multispecies quantitative approach applied to Bolognese sauce. Food Control.

[44] Ma X., Li H., Zhang J., Huang W., Han J., Ge Y., Sun J., Chen Y. (2020). Comprehensive quantification of sesame allergens in processed food using liquid chromatography-tandem mass spectrometry. Food Control.

[45] Montowska M., Fornal E., Piątek M., Krzywdzińska-Bartkowiak M. (2019). Mass spectrometry detection of protein allergenic additives in emulsion-type pork sausages. Food Control.

