# Supplementary material for: Multispecies Identification of Oilseed- and Meat-Specific Proteins and Heat-Stable Peptide Markers in Food Products
Source: Molecules. 2021 Mar 12;26(6):1577. doi: 10.3390/molecules26061577 (PMC7998630; doi:10.3390/molecules26061577)
Supplement: Supplementary file 1 [file molecules-26-01577-s001.pdf]

Supplementary Material

Article

Multispecies identification of oilseed- and meat-specific proteins and heat-stable peptide markers in food products

Klaudia Kotecka-Majchrzak [1], Natalia Kasałka-Czarna [1], Agata Sumara [2], Emilia Fornal [2] and Magdalena Montowska [1,*]

[1] Department of Meat Technology, Poznan University of Life Sciences, Wojska Polskiego 31, Poznan 60-624, Poland; klaudia.kotecka@up.poznan.pl; magdalena.montowska@up.poznan.pl
[2] Department of Pathophysiology, Medical University of Lublin, Jaczewskiego 8b, Lublin 20-090, Poland; agatab9419@gmail.com; emilia.fornal@umlub.pl
* Correspondence: magdalena.montowska@up.poznan.pl; Tel.: +48 61 848 7257

Table S1. Peptides unique to oilseed proteins analysed in commercial food products (coconut, flaxseed, hemp seeds, milk thistle seeds, nigella seeds, pumpkin seeds, rapeseed, sesame seeds, sunflower seeds).

Protein	Parent Ion (m/z)	Exp z	Peptide Marker	P1	P2	P3	P4	P5	P6	P7	P8	P9	P10	P11	P12
**coconut**															
11S globulin isoform 2 (AKS26849.1)	991.5071	2	GFGTELLAAAFGIDMELAR		-							-		-	
	635.3315	2	AGSEGFQFVSIK		-							-		-	
	561.7771	2	GETVFDGELR		-							-		-	
	714.3377	3	GMVGLVMPGCPETFQSFQR		-							-		-	
	523.7641	4	GRVEVADDKGETVFDGELR		-							-		-	
cocosin (ASQ40963.1)	619.6739	3	QGQLLIVPQNFAMLER		-							-		-	
	681.9973	3	AENGLQVLRPSGMEEEER		-							-		-	
	432.2241	2	CAGVSTIR		-							-		-	
	1011.4302	2	SEAGVTDYFDEDNEQFR		-							-		-	
alpha galactosidase isoform 2 (AIL28756.1)	1051.9654	2	LGYQYVNLDDCWAESNR		-							-		-	
	1112.5950	2	EILINEEAIAVNQDALGVQGK		-							-		-	
	776.3615	2	LGIYGDAGFYTCSK		-							-		-	
	1289.1257	2	GSSQTSITANWSDIGLDPSTVVDAR		-							-		-	
	903.0668	3	TPQMGWNSWNHFACNIDEQMIK		-							-		-	
	581.6339	3	VKGDGSAEVWAGPLSGGR		-							-		-	
oleosin isoform 500a (AQT25673.1)	456.5737	3	RPPGSEQLEQAR		-							-		-	
oleosin isoform 300a (AQT25672.1)	487.5738	3	HHQALPAAAMEER		-							-		-	
Oleosin isoform OLE500c partial (ACH91013.1)	599.3204	2	VPGAEQLEQAR		-							-		-	
**flaxseed**				**P1**	**P2**	**P3**	**P4**	**P5**	**P6**	**P7**	**P8**	**P9**	**P10**	**P11**	**P12**
hypothetical protein (AND01131.1) / tripeptidyl peptidase II (AFN53692.1)	625.8470	2	DIPIFQYNIK		-										
	569.2937	2	DQLAEALYSK		-										
	873.4648	2	FIEVPLGASWAEATVR		-										
	668.3549	3	LSLLEEIGWTHLTAYEK		-										
	553.2980	2	SFNLVVDPSK		-										
	363.5376	3	TLEDKDVIR		-										

	678.3460	2	VYSVGDVYPAASK		-										
allene oxide synthase (P48417.1)	434.7484	2	AGLELHTK		-										
	635.3257	2	EVEEATLHSVR		-										
	488.9379	3	LPPLLVQNDYHR		-										
	826.4018	3	LYEFFTSAAGSVLDEAEQSGISR		-										
conlinin (CAC94011.1)	658.3083	2	DLPGQCGTQPSR		+										
	646.9305	3	GGGQQSQHFDSCCDDLK		+										
	745.3280	2	GGQGGQGQQQQCEK		+										
	857.4147	2	QDIQQQGQQQEVER		+										
	596.8029	2	QIQEQDYLR		+										
oleosin high molecular weight isoform (ABB01624.1)	808.3767	2	GGPYHQQGTGSGPSASK		-										
	600.2646	2	MQDAAGYMGQK		+										
	862.0914	3	TTQPHQVQVHTQHHYPTGGAFGR		-										
	722.0281	3	YLQQAGQGVGVGVPDSFDQAK		-										
conlinin (CAC94010.1)	983.3842	2	GGGEQSQYFDSCCDDLK		+										
	888.0736	3	GGGQGGQGQQQSCEQQIQQQDFLR		+										
	863.9276	2	QEIQQQGQQQEVQR		+										
	541.7418	2	QLSTGCTCR		+										
	622.2671	2	SCQQFMWEK		+										
chitinase IV, partial (ABA39179.1)	758.3822	2	ANGFDGVANPDIVAR		-										
	356.1756	3	SRYPCAQGK		-										
late embryogenesis abundant (lea) (AMY26620.1)	494.2600	2	AVAQEQVDK		-										
	844.0464	3	QGEQVTGGYGNYTTGGQTGYNHQI		-										
	442.2267	2	VSAHGPMGK		-										
chain A, Linum usitatissinum trypsin inhibitor (P82381.1)	507.2754	2	NAWPELVGK		-										
	553.7683	2	SGNMAAATVER		-										
	986.5374	2	VWVIVNDHGVVTSVPHIT		-										
amaranthin-like lectin (AIU47287.1)	636.3531	3	LQVVPSTIDPTSMIHLR		-										
	424.2331	3	SLEAHTVLSWK		-										
oleosin, partial (ABB01620.1)	795.8659	2	ASDFGQQHVTGQQTS		-										
oleosin isoform (ABB01617.1)	838.3924	2	ASEFAQQHVTGGQQTS		-										
oleosin isoform (ABB01618.1)	831.3817	2	ASEFGQQHVTGGQQTS		+										

hemp seeds				P1	P2	P3	P4	P5	P6	P7	P8	P9	P10	P11	P12
edestin 1 (CDP79023.1)	597.0566	4	NAIYTPHWNVNAHSVMYVLR								+	+	+		
	822.3950	2	YLEEAFNVDSETVK								+	+	+		
	930.8344	3	YTIQQNGLHLPSYTNTPQLVYIVK								+	-	+		
	749.6201	4	VEAEAGLIESWNPNHNQFQCAGVAVVR								+	+	+		
	708.6764	3	GILGVTFPGCPETFEESQR								+	+	+		
	715.3259	2	GQGQGQSQGSQPDR								+	+	+		
	745.4229	2	ISTVNSYNLPILR								+	+	+		
	744.3454	2	QASSDGFEWVSFK								+	+	+		
	380.5400	3	VQVVNHMGQK								-	+	+		
	705.3615	2	EETVLLTSSTSSR								+	+	+		
	473.2245	2	LQGQNDDR								+	+	-		
	535.8186	2	GTLDLVSPLR								+	+	+		
	397.1866	3	QQNQCQIDR								+	+	+		
edestin 2 (CDP79028.1)	562.9650	3	ILAESFNVDTELAHK								+	+	+		
	824.4140	2	AMPDDVLANAFQISR								+	+	+		
	763.7034	3	NGMMAPHFNLDSHSVIYVTR								+	+	+		
	756.4102	3	GLLLPSFLNAPMMFYVIQGR								+	+	+		
	653.8455	2	ASAQGFEWIAVK								+	+	+		
	434.8582	3	SEGASSDEQHQK								+	+	+		
	779.4463	2	LNTLNNYNLPILR								+	+	+		
	527.5920	3	DEISVFSPSSQQTR								+	+	+		
	634.2869	2	WQSQCQFQR								+	+	+		
	508.2638	2	LQVVDDNGR								+	+	+		
	400.2213	3	GEDLQIIAPSR								+	+	+		
edestin 3 (SNQ45160.1)	783.6132	4	VECEGGMIESWNPNHEQFQCAGVALLR								+	+	+		
	593.2834	3	FYIAGNPHEDFPQSR								+	+	+		
	847.4157	2	AMPEDVIANSYQISR								+	+	+		
	1049.0167	2	GFSVNLIQEAFNVDSETAR								+	+	+		
	894.5037	3	LTIQPNGLHLPSYTNGPQLIHVIR								+	+	+		
	834.3774	2	TAVYGDQNECQLNR								+	+	+		
	846.7509	3	GVLGTLFPGCAETFEEAQVSVGGGR								+	+	+		
	440.8728	3	SRQEQEQEMR								-	+	+		
	583.7990	4	NAMYAPHYNINAHSIIYAIR								+	-	+		
	376.1713	3	LEACEPDHR								+	-	+		

	800.4476	2	QGQALTVPQNFAVVK								+	+	+		
	894.5037	3	LTIQPNGLHLPSYTNGPQLIHVIR								+	+	+		
	823.0491	3	FYIAGNPHQEFPQSMMTQQGR								+	-	+		
edestin 3 (SNQ45158.1)	823.0491	3	FYIAGNPHQEFPQSMMTQQGR								+	-	+		
	376.1713	3	LEACEPDHR								+	-	+		
7S vicilin-like protein (SNQ45153.2)	690.3825	3	TLFLPQYLDSELTIFIR								-	-	-		
	1016.0237	2	EILSSQQEGPIVYIPDSR								-	-	+		
	715.8861	2	GPELAAAFGLSLER								+	-	+		
	711.3414	3	NNYGWSIALDEFSYSPLR								-	-	-		
	690.3823	3	TLFLPQYLDSELTIFIR								-	-	-		
**milk thistle seeds**				**P1**	**P2**	**P3**	**P4**	**P5**	**P6**	**P7**	**P8**	**P9**	**P10**	**P11**	**P12**
preprosilpepsin 2 (AGE15495.1)	772.361	3	NVNEEEGGELVFGGVDPNHFR												-
	764.4089	2	IFELTPEQYIFK												-
**nigella seeds**				**P1**	**P2**	**P3**	**P4**	**P5**	**P6**	**P7**	**P8**	**P9**	**P10**	**P11**	**P12**
chain A, nigellin-1.1 (PDB: 2NB2_A)	775.3697	2	ACIGLCAPACLTSR							+					
	716.2870	2	YQDCLSECNSR							+					
	568.2364	3	DRYQDCLSECNSR							-					
	673.7993	2	CTYIPDYAGMR							+					
thionin NsW1 (C0HJH9.1)	521.7097	2	TCSGLCGCK							-					
**pumpkin seeds**				**P1**	**P2**	**P3**	**P4**	**P5**	**P6**	**P7**	**P8**	**P9**	**P10**	**P11**	**P12**
11S globulin subunit beta-like (XP_023553731.1)	1040.4737	3	IEAEGGVIEMWDPNHEEFQCAGVAFQR	+							+				
	1039.4171	2	GDEQQWEEEQEEEQER	+							+				
	810.3756	3	GVVLSGCPETYQESQQSAGEFR	+							+				
	786.9306	2	VLAEVLNINTEMAR	+							+				
	466.9336	3	VEGQFEVIKPPR	+							+				
	390.1927	3	SEYKQEQTR	+							+				
	604.0913	4	LIIVVLLDVSNHANQLDFHPR	+							-				
11S globulin subunit beta (XP_023515280.1)	882.9373	2	GIAIPGCAETYQTDLR	+							+				
	776.3399	4	AEAEAGFTEVWDQDNDEFQCAGVNMIR	+							+				
	697.3758	2	MLPLGVLSNMYR	+							+				
	742.9190	2	ISTANYHTLPVLR	+							+				
	923.4489	3	GVLYSNAMVAPHYTVNSHSVMYATR	+							+				
	906.7749	3	SGNLFSGFADEFLEEAFQIDGGLVR	+							+				
	405.2375	3	GLLLPGFSNAPK	+							+				

	603.8593	2	LVFVAQGFGIR	+							+				
	715.8913	2	NVANQIDPYLRK	-							+				
	488.9258	3	FYLAGRPEQVER	+							+				
11S globulin seed storage protein 2-like (XP_023553732.1)	999.1273	3	IESEGGITELWDEAEEDFQCAGVAAIR	+							+				
	964.4567	3	LVYVVDGEANFQISDDYGNQVFNER	+							+				
	623.3219	2	SPIAGYTSFFR	+							+				
	920.0450	3	CDEQMSFLTPEEEEEELSESPSR	+							+				
	807.8990	4	NNDLVNIYNGFDQDFLAQAYNVPTDLVR	+							+				
	574.6158	3	QHTFLFPPSSGSSSSR	+							-				
	1039.8593	3	GNMFVIPQFYPALVQAGQEGFEWITFK	+							+				
vicilin-like (XP_023527143.1)	1045.1785	3	DYLSAGGEAQAYYSVFSNDVLEAALNIPR	+							+				
	885.8120	3	LSEGGVLVIPAGHPIAIMASPNENLR	+							+				
	1030.9535	2	TEQEQSNNPYYFQEQR	+							+				
	709.3653	2	LVGFGINAENNNR	+							+				
	671.3642	2	LVQPVNNPGEFK	+							+				
	1214.5876	3	ESYNVESGDVMTIPAGTTLYLANQENEDL QIVK	+							+				
	545.3144	2	ATITTVVQEK	+							+				
	383.2134	3	TDVATSVVDIK	+							+				
11S globulin seed storage protein 2-like (XP_023532597.1)	1010.4435	3	IESEGGISEIWDESEEEFQCAGVAAMR	+							+				
	1214.1502	2	ALPQQVLEQSFQITAGEAQQLK	+							+				
	409.2258	3	SPVVGYTSLFR	+							+				
	462.0336	5	GHLFPNAQYNLHWSITDHR	+							-				
	1012.1700	3	GNMFVIPQFYASLAQAGPEGFEWVTFK	+							-				
	483.9112	3	HTFLFPPSSSSSR	+							-				
	1091.8718	3	SSEDLVNIFFGFDQELLAEAYNIPSDLAR	+							-				
vicilin-like seed storage protein (XP_023511963.1)	911.4301	2	SPAVASAFDITEDDLDR	+							-				
seed biotin-containing protein SBP65-like (XP_023534218.1)	806.9187	2	SLQQGVEAGTGEAIPR	-							-				
	1200.0409	2	GTEEAEDTVEEGNQYTGLETVK	-							-				
	553.9748	3	GAAEYAGAIAAKPLSAAK	-							-				
	426.7374	4	VPQLASHFEAIAVHGK	+							-				
	789.3820	2	QNQPSLEEISNYR	-							-				
	567.7857	2	NVAESTGETVK	-							-				
	636.3088	4	QGVGGEKPEDSHELAAQFESLADK	-							-				

11-beta-hydroxysteroid dehydrogenase 1B-like (XP_023519948.1)	823.4285	2	ILAMPAAGASESDALTK	+						+					
	728.1320	4	LDHLVNNAAITHLVMFEDIADIAAFR	+						-					
	788.9386	2	IVALSAPPAWMPAPR	+						-					
	586.3120	3	DALIGLVPVETAEACAK	+						+					
	686.3616	2	ENLLEEVADIAR	+						-					
	445.8719	3	VMNETMNHFGR	+						+					
sucrose-binding protein-like (XP_023547904.1)	495.0059	4	VPSGAPFYFINKDEHQK	+						-					
	683.6883	3	IWPFSEGDTERPFNLLK	+						-					
	729.8218	2	QSFDVECGDVFR	+						+					
embryonic protein DC-8-like (XP_023527803.1)	730.0488	3	GGDTVVVGMEETRPGALASTLK	+						-					
	539.2632	2	EATGDTVASAR	+						-					
enolase 1-like (XP_023518286.1)	619.3318	3	GNPTVEVDLVLSDGTLAR	-						-					
late embryogenesis abundant protein D-34-like (XP_023548951.1)	959.4613	2	DAATMQAAENMLLGQTPR	-						-					
	669.6792	3	YGDVFDVSGDLASKPIAPR	+						-					
	800.7315	3	ATGNNEITPGGIGAEAQSAASLNTR	-						-					
	979.9830	2	NDPTMTTTPGGVAASVAAAAR	-						-					
late embryogenesis abundant protein 7 (XP_023548700.1)	563.7489	2	TSQAMGTMGDK	+						-					
	566.2366	2	DQTGSYMSDK	+						-					
	409.2051	3	EKTYETGQAAK	-						-					
protein disulfide-isomerase-like (XP_022939823.1)	796.4248	3	LAPTLEEVAVSYESDPEIIIAK	-						-					
	959.8156	3	FIEESSIPLVTVFDNDPNNHVYLSK	-						-					
1-Cys peroxiredoxin A (XP_023539587.1)	662.9924	3	LIGLSCDDVQSHTDWIK	-						-					
2S albumin (XP_023545481.1)	680.2948	3	NVDEECRCDMLEEIAR	+						+					
	476.9158	3	NLPSMCGIRPQR	+						+					
	568.7618	2	CDMLEEIAR	+						+					
alpha-xylosidase 1-like (XP_023549718.1)	830.9387	2	GLSLPVGNNFEISWK	-						-					
	719.0280	3	LPEDSSLYGLGENTQPHGIK	-						-					
peroxygenase-like (XP_023547968.1)	545.6017	3	LSLGELWDMTEANR	+						-					
	815.1044	3	TQQGWIPSPFLPIYIYNIHR	+						-					
	544.2997	3	NDLETTIPKPYLAR	-						-					
	472.2696	4	VRNDLETTIPKPYLAR	-						-					
dehydrin DHN3-like	719.3201	3	TGTGTGTGMYEGGGGGQQQQQR	-						-					

(XP_023553197.1)	753.3639	3	QTDEYGNVISDTAQHVDPLR	+							-				
	673.5634	4	GGMFHHDKPVSTGGSVGYTTGEQGER	+							-				
17.1 kDa class II heat shock protein-like (XP_022941228.1)	833.4392	2	VSAAYQDGVLSVTVEK	+							-				
	933.7846	3	AMAATPADVVEYPNAYQFSIDMPGLK	-							-				
	800.4212	2	VQIEDNQLVVTGER	+							-				
2S albumin-like (XP_023520423.1)	669.3198	3	AHEEIGSCVQYLTQQSR	+							+				
	935.9260	2	NLPSMCGLRPQQCYF	+							+				
11 kDa late embryogenesis abundant protein-like (XP_022940345.1)	823.9102	2	TGAHTGLTTGTGTGTGTR	+							-				
	516.7579	2	ESAANVGASAR	+							-				
oil body-associated protein 1A-like (XP_023549320.1)	804.0800	3	SEIPGEATHASTAVLETATAAVQR	+							-				
	920.0151	2	GDILPLGIPQLMMALTR	-							-				
	766.4016	2	SGTLFMPGVPGAIER	+							-				
cysteine proteinase inhibitor 12-like (XP_023548422.1)	609.3421	3	SNSLLPYELLEIVHAK	+							-				
	896.9125	2	NSEGNFLLNQMVPDQS	-							-				
oleosin 18.2 kDa-like (XP_023550995.1)	621.2876	2	MQEMAGYVGQK	+							+				
	509.2526	2	EAGQEIQSR	+							+				
	504.2627	5	QVQVHHQQQRPSYLQEPTWK	-							-				
oleosin 18.2 kDa-like (XP_022928435.1)	632.8127	2	TTTTTAAQEQGR	+							+				
17.4 kDa heat shock protein (XP_022939316.1)	766.3900	2	VPSSGAGETTAIANTR	+							-				
**rapeseed**				**P1**	**P2**	**P3**	**P4**	**P5**	**P6**	**P7**	**P8**	**P9**	**P10**	**P11**	**P12**
cruciferin CRU4 (XP_013585668.1)	909.1354	3	GSIHNNAMVLPQWNVNANAALYVTK	-		-		-		-	-				
	1011.0350	2	QNNIFNGFAPQILAQAFK	-		-		-		-	-				
	1252.5595	3	VTPGCAETFMDSPVFGQGQGQEQGQGQGQGQGQGFR	-		-		-		-	-				
	1082.2884	4	CGDTIATPPGVAQWFYNNGNEPLILVAAADIANNLNQLDR	-		-		-		-	-				
	353.1999	4	LTFVVHGHALMGK	-		-		-		-	-				
	1239.2795	3	CTENLDDPSSADVYKPSLGYISTLNSYNLPILR	-		-		-		-	-				
	687.3447	2	SNDNAQINTLAGR	-		-		-		-	-				

	754.3585	3	FSTLETTLTQSSGPMGYGMPR	-		-		-		-	-				
jacalin-related lectin 36-like (XP_013672510.1)	929.1074	3	TLLGSEVFEVDPDDYITSAEVQSDR	-		-		-		-	-				
	885.4617	2	IFGQDTDVITCLIFK	-		-		-		-	-				
	711.3682	2	TTGNEDAIAALAFK	-		-		-		-	-				
	746.6984	3	AGEVLHALGAYFSPSSSTSSGGR	-		-		-		-	-				
	597.3249	2	VQVTYEDVIK	-		-		-		-	-				
	1116.5400	4	DNQIAGFQGISSNVLNSIDVHFAPLPSSSTSDSSTPSSASQANK	-		-		-		-	-				
	662.8210	2	TSSSTQTAGSAAGAK	-		-		-		-	-				
	424.2241	3	TQNQFSIHAPK	-		-		-		-	-				
embryonic protein DC-8-like isoform X1 (XP_013687874.1)	788.8919	2	AGTVGWTAAHFTTEK	-		-		-		-	-				
	792.0809	3	YAQEQGAGAGGVLGAIGETIAEIAK	-		-		-		-	-				
	392.2013	3	GAAHYTGVAAEK	-		-		-		-	-				
	706.8731	2	TTTNIVIGDPPER	-		-		-		-	-				
	427.2050	3	MTSHFESIAEK	-		-		-		-	-				
	539.7549	2	GGSVQDTASEK	-		-		-		-	-				
	481.7617	2	EQTVSVTAK	-		-		-		-	-				
	575.2996	2	ATLEDIQGFR	-		-		-		-	-				
myrosinase (AAV80206.1)	532.2624	2	GYAVGTDAPGR	-		-		-		-	-				
	675.8510	2	GEFLGPLFVEDK	-		-		-		-	-				
	450.8870	3	GVNQGGLDYYHK	-		-		-		-	-				
nudix hydrolase 3 (RID54770.1)	1039.5188	2	YSPVTLEANLAELSEADQK	-		-		-		-	-				
oleosin S2-2-like (XP_013677557.1)	859.8713	2	VGYGAGYDYGYGADYK	-		-		-		-	-				
	784.8692	2	AHEAHDTSLTTETR	-		-		-		-	-				
17.6 kDa class II heat shock protein-like (RID42282.1)	785.7318	3	FPIISILEDMLEVPEEHSEK	-		-		-		-	-				
napin-3: 1.7S seed storage protein (P80208.1)	927.4855	2	QQQGQQGQQLQQVISR	-		-		-		-	-				
major oleosin NAP-II (RID78718.1)	579.9246	3	AQYYGQQQTGGEHDR	-		-		-		-	-				
	414.1971	3	DRDQYSMIGR	-		-		-		-	-				
	523.7233	2	DQYNMYGR	-		-		-		-	-				
peroxidase 12	720.8657	4	NLDITDLVALSGGHTIGIAHCPSFTDR	-		-		-		-	-				

(XP_009105826.1)															
napin small chain S2 (pir||S70337)	537.2426	2	QAMQPGGGSGPS	-		-		-		-	-				
**sesame seeds**				**P1**	**P2**	**P3**	**P4**	**P5**	**P6**	**P7**	**P8**	**P9**	**P10**	**P11**	**P12**
legumin B-like (XP_020549903.1)	952.0771	3	GMYGVMISGCPETFESSQQQFEEGR				-								
	769.5992	4	IQAEGGVSEFWDHNSDEFQCAGVSIHR				+								
	691.9869	3	GESDMQIVNHNGQAVFDGR				+								
	704.0222	3	GFDVQILSEVFGVDEQTAR				+								
	932.4297	3	AGEQGCEWVEFNTNDNALINTLSGR				-								
	742.7351	3	ALMLPAYHNAPILAYVQQGR				+								
	764.7719	3	FSTINSLTLPILSFLQLSAAR				+								
	775.7190	3	NGIMAPHWCVNAHSVIYVTR				-								
	752.3649	2	GQEQQEYAPQLGR				+								
	524.2414	2	SLQGENDER				-								
	794.4355	2	GLPADVIANAYQISR				+								
	397.2029	3	GQHQFGNVFR				+								
	604.2730	2	GSTWQQGQCR				-								
11S globulin seed storage protein 2 precursor (NP_001291336.1)	805.7626	3	AMPLQVITNSYQISPNQAQALK				+								
	717.3644	3	AFDAELLSEAFNVPQETIR				+								
	927.7903	3	GNLYSNALVSPDWSMTGHTIVYVTR				+								
	769.3669	2	QEQFQCAGIVAMR				+								
	689.6622	3	GDAQVQVVDHNGQALMNDR				+								
	497.9773	4	MTFVRPDEEEGEQEHR				+								
	354.8553	3	QTFHNIFR				+								
	502.2730	4	STIRPNGLSLPNYHPSPR				-								
	670.3274	2	AGNNGFEWVAFK				+								
legumin B-like precursor (NP_001291327.1)	738.0355	3	NGITAPHWSTNSHSALYVTR				-								
	811.8886	2	AMPDEVVMNAFGVSR				+								
	1049.4783	3	LQSEAGVTEFWDANNEEFQCAGIEFVR				-								
	1034.0010	2	FFLAGNPQTAQFQGQQER				+								
	667.3341	2	TECQVQQLTAR				-								
	777.3863	3	EGVTHWAYNDGDTPIISVSIR				+								
	702.3340	2	AGEQGFEYVTFR				+								
	880.1456	3	SVLNEEVNEGQLVVVPQNFALAIR				+								
	410.4540	4	YWQSLQQHQQHR				+								

	595.3488	3	ISTINSQTLPILSQLR				+								
	568.2753	2	DEATVFSPGGR				+								
vicilin-like antimicrobial peptides 2-2 (XP_011090693.1)	761.9109	4	LAVLEANPNTFVLPHHFDAESVLVVAGG K				-								
	644.3726	2	VPAGSIVYLVNR				-								
	647.3257	2	SELLQGVDNYR				-								
	686.3061	2	VYEQCLSQCGK				-								
	1137.2876	3	LSIGDAFIVPAGHPIAMIASQDSNLQLVSF GIK				-								
	607.9527	3	QEQSYFLPGPGQGEER				-								
	975.9475	2	YGQYFEASPNDYQQLK				-								
	726.3638	2	DLDVSVGFMNINK				-								
	1056.5059	2	YFLAGQDNIWNQVESEAK				-								
	750.0434	3	EESWGPFNLLEGRPLFSNR				-								
embryonic protein DC-8-like (XP_011098036.1)	903.9173	2	IQGGETFTSTEQHDTR				-								
	840.3983	2	DAVVATEEQGAEYAAR				-								
	586.6379	3	GQAVVAGWGAAHYTLEK				-								
	567.6463	3	DLLVGQYPAQVIEQK				-								
	704.8576	2	VASAGQTVAGYAGEK				-								
	482.2371	3	GEQDVAHPLHGER				-								
vicilin-like antimicrobial peptides 2-2 precursor (NP_001291343.1)	761.3528	3	IALVVDGEGYFEMACPHMSR				-								
	753.1482	4	VAILEAEPQTFIVPNHWDAESVVFVAK				-								
	963.4937	4	GTVVIIPAGHPFVAVASSNQNLQVLCFEV NANNNEK				-								
	490.2204	2	EACIQACK				-								
poly [ADP-ribose] polymerase 3 (XP_011088879.1)	936.0066	2	VANSVIGATCLVVSPVER				-								
	802.8999	2	ETQPLDAFNIVSDR				-								
	459.2513	2	DSGVADLLK				-								
	596.2985	2	EAWLSESIEK				-								
	930.9576	2	VTELTSPPENSTSLEEK				-								
vicilin-like seed storage protein At2g28490 (XP_011089873.1)	922.4365	2	IEAGSAFYLMNTGEGQR				-								
	892.4148	3	KPDFQNNYGWSTALDESDYSPLK				-								
	535.8019	2	LNEIINAQR				+								
	689.2950	2	WEGGGEEGGTGWR				-								
glucose and ribitol	906.7762	3	IDILVNNAAEQYEASTVEEIDEPR				-								

dehydrogenase homolog 1-like precursor (NP_001291332.1)	756.7043	3	EHIMDPTPQATTPEYKPANK				-								
	691.8411	2	TNIFSYFFTSR				-								
oil body-associated protein 2A-like (XP_011102222.1)	670.8246	2	GFAIEVESTEMK				-								
	746.3968	3	LPMGPPALMMSPQELDLGIVK				-								
	803.4257	3	LIGVEYIISGGIFESLSPEEQK				-								
protein disulfide-isomerase-like (XP_011099696.1)	712.3827	4	NAFQYFGLEVEQAPLLIIQKPDGQK				-								
	849.1275	3	QLAPILEEVAVHFENDPNVVIAK				-								
aspartic proteinase-like (XP_011096039.2)	768.0265	3	NVEEEEGGELVFGGVDPNHFK				-								
	773.0764	3	AVVEQYGQTIMDLLLAEAQPK												
steroleosin (AAL09328.1)/ 11-beta-hydroxysteroid dehydrogenase 1B-like (NP_001291322.1)	806.8865	2	LFCPEVMEWVFR				-								
	800.4357	2	SLLYPETVQVPEPK				-								
	667.8377	2	LMYLASPGEPEK				-								
	614.8488	2	DVQVSTTPILR				-								
	549.6250	3	IVVLSSSSSWMPTPR				-								
	578.3271	2	DLGSPDVVVVR				-								
malate synthase, glyoxysomal (XP_011073010.1)	590.3035	5	QCTTPVLDDFLTLDAYNHIVIHHPK				-								
	602.3274	2	DAVQFVAGLQR				-								
glucose and ribitol dehydrogenase (XP_011090772.1)	636.9817	3	EHLMDPIPQALNPDYR				-								
	624.3235	2	VVDEVVNNFGR				-								
peroxygenase (NP_001291323.1)	751.8715	2	YLPMNFENLFSK				-								
	697.0465	3	NAALAPDAPLAPVTMERPVR				-								
embryonic protein DC-8 isoform X1 (XP_011099627.1)	814.3645	2	NAYESASETASDVAGR				-								
	648.2934	2	AMESASDIASDAK				-								
	574.2671	2	AYDVYSSASGK				-								
	584.2641	2	DMSSDLASDVK				-								
	592.2496	2	VNMAEDMSMR				-								
	541.2421	3	MNEAADQAASMAGQAR				-								
embryonic protein DC-8 (XP_011101471.1)	983.4937	3	LTVDEEGTPVVMVVDVDETTAGATASTLK				-								
	638.2663	2	DATMETMGEYK				-								
	558.5986	3	LMPHATETTEETGTR				-								
1-Cys peroxiredoxin	955.9831	2	LLGVSADDVQSHNEWIK				-								

(XP_011100693.1)	597.2952	3	QMFPQGYQTAHLPSGK				-								
	608.8204	2	VNYPIAADPTR				-								
	655.5993	4	IATPANWKPGDPVVISPTVSNQEAK				-								
vicilin-like seed storage protein At2g18540 (XP_020554796.1)	747.4023	2	ALVSTEYGQISAVR				-								
	812.4227	3	VPEEVIDELFNGTQQPTIIHR				-								
	1098.0518	2	LPQGSVFFIQSNLETETER				-								
18.8 kDa class II heat shock protein (XP_011081635.1)	917.9424	2	FALPENANTEAISAACR				-								
	905.4338	4	NFGLDNSLLSTLQDMLDFAEDHEKPTQNNPSK				-								
	759.3521	3	EYPSSYVFMVDMPGIAGGDIK				-								
osmotin-like protein (XP_011081307.1)	527.6124	3	ADLLSTCPHALQVR				-								
glyoxysomal fatty acid beta-oxidation MFP-a (XP_011071437.1)	862.7622	3	FCPPHCILGSNTSTIDLNLIGEK				-								
late embryogenesis abundant protein Dc3 (XP_011072248.1)	454.4482	4	HTFGMADVDEEDPHSK				-								
aldose reductase (XP_011095083.1)	817.9529	4	VRPALLNTLGELQLDYLDLYLIHWPFR				-								
	408.8735	3	DLIHDPMVER				-								
UTP--glucose-1-phosphate uridylyltransferase (XP_011101235.1)	599.8381	2	LEIPDGSVIAGK				-								
	1000.0223	2	VVVPYDSLAPVPEDAAQTK				-								
7S globulin (AAK15089.1)	614.7641	2	GCEQQHGEQR				-								
	623.6509	3	IPYVFEDQHFITGFR				+								
	982.1529	3	LLQPVSTPGEFELFFGAGGENPESFFK				+								
	1003.8619	3	VAILEAEPQTFIVPNHWDAESVVFVAK				+								
2S albumin precursor isoform 3 (ABB60053.1)	655.7817	2	EQEMQQMMQK				+								
	695.7730	2	MCGMSYPTQCR				+								
**sunflower seeds**				**P1**	**P2**	**P3**	**P4**	**P5**	**P6**	**P7**	**P8**	**P9**	**P10**	**P11**	**P12**
11S globulin seed storage protein 2-like (XP_022035002.1)	715.4037	2	LPILSLMDLSAEK		-	+	+	+	+		+		-		
	869.1251	3	NQQVVAVIVDDVNNPANQLDLQAK		-	+	-	+	+		+		-		
	809.4145	3	AMPLEVISYSYQVSPSQAQSLK		-	+	-	+	+		+		-		
	660.3401	2	AGQNGLEWVAFK		-	-	-	+	+		+		-		

	720.7065	3	MTLQHNSLFLPSFQPFPR		-	+	-	+	+		+		-		
11S globulin seed storage protein G3-like (OTG20713.1)	973.4912	2	VQIVNNQGNSVFDNELR		-	+	+	+	+		+		-		
	518.2806	3	FPILEHLQLSAER		-	+	+	+	+		+		-		
	660.9787	3	ENIDNPSHADFVNPQAGR		-	-	-	+	+		+		-		
	528.5540	4	GHIVNVGQDLQIIRPPQAR		-	+	+	+	+		+		-		
	619.6456	3	FFLAGNPQAQSQQQHR		-	+	+	+	+		+		-		
	1059.8220	3	GIQGVILSGCPETYEYSQEQQFSGQSER		-	+	+	-	+		+		-		
	627.3320	2	ETVLFAPSFSR		-	+	+	+	+		+		-		
seed biotin-containing protein SBP65-like (XP_022022103.1)	636.6244	3	VEETMYGTAGGGIHDQSR		-	-	-	+	-		-		-		
	819.8936	2	NIAMETGQTAAEFAGK		-	-	-	+	-		-		-		
	601.3265	2	ALGVAETAVDAGK		-	-	-	+	+		-		-		
	820.3820	2	AAESAAEFASDTSAGVR		-	-	-	-	-		-		-		
	630.9920	3	AAGVATDAAVTGTNIAMATGK		-	-	-	+	-		-		-		
	693.3553	3	TAAELATGAAVTGTNIAMETGK		-	-	-	+	-		-		-		
11S seed storage protein globulin subunit beta-like (OTG06044.1)	924.7816	3	FFLAGNPQSQEQQGEPWGQSQRPR		-	-	-	+	+		+		-		
	702.7277	3	GLLLPYYPNTPELVYVVR		-	+	-	+	+		+		-		
	854.9328	2	ALPEEVLMNSYSISR		-	-	-	+	+		+		-		
	525.2605	4	TGQSQRPGWETGRPEQQR		-	-	-	+	+		+		-		
	786.4138	2	ANIASPAIADVYNPR		-	+	-	+	+		+		-		
	615.2728	3	EEQEWEQYHPGQQR		-	-	-	+	+		+		-		
	696.8315	2	TNDNAMTTQLAGR		-	-	-	+	+		+		-		
	592.0920	5	SGQTQRPSWETGRPGQSQRPSWETGR		-	-	-	+	-		-		-		
	744.9083	2	LPVLESFQLSAER		-	+	-	+	+		+		-		
	1026.2117	3	EGDILALPAGAVHWTYNEGDTPLVVVAL R		-	+	-	+	+		+		-		
	793.7142	3	GQGLQGTVLPGCVETFETSEGGR		-	-	-	+	+		+		-		
	762.3873	4	NDGSQVFDDWVQEGQLIVVPQDFAVIK		-	-	-	+	+		+		-		
seed storage albumin 2 precursor (ALO17641.1)/ hypothetical protein HannXRQ_Chr11g03377 91 (OTG08095.1)	654.8052	2	GQFGGQEMDIAR		+	+	+	+	+		+		-		
	756.9030	2	AQILPNVCNLQSR		+	+	+	+	+		+		-		
	741.0084	3	SQQCSETEIQRPVSQCQR		+	+	+	+	+		+		-		
	493.5518	3	ECQCEAVQEVAR		+	+	+	+	+		+		-		
	504.2586	2	MFLQQGQR		+	+	+	+	+		-		-		
	815.7251	3	VIQNLPNQCDLEVQQCNIPY		-	+	-	+	+		+		-		
	1035.9670	4	GQQHQQQQEQQLLQQCCQELQNIDQQC QCEAVK		-	+	-	+	+		-		-		

	885.4207	2	YVEQQMQSPMPYIR		-	-	-	+	-		-		-		
	754.0147	3	QQEQQGLQQCCNELQNVR		-	-	-	+	+		-		-		
	609.7864	2	QCSQQVQGQR		-	-	+	+	+		+		-		
protein disulfide-isomerase-like (OTF94178.1)	935.8423		FIVDSSIPLVTLFDQSPTNQPFLVK		-	-	-	+	-		-		-		
	787.4077	3	LAPILDEVAVSFENDADVMIAK		-	-	-	-	-		-		-		
	630.8015	3	TPEDAGSLIDDK		-	-	-	-	-		-		-		
	567.2946	2	GESSVTTPTVR		-	-	-	+	-		-		-		
embryonic protein DC-8-like (XP_022038548.1)	667.3469	2	ISEGLGLSTEEAK		-	-	-	-	-		-		-		
	563.2754	2	DAATSAAEYVK		-	-	-	-	-		-		-		
aconitate hydratase cytoplasmic (OTG31512.1)	878.7623	3	DVWPSTEEIAEVVQSSVLPDMFR		-	-	-	-	-		-		-		
	735.3841	2	VVSFSFGGQTAELK		-	-	-	-	-		-		-		
malate synthase glyoxysomal (OTG26019.1)	913.9604	2	EDAANITEEDLLQIPR		-	-	-	-	-		-		-		
embryonic protein DC-8-like (OTG01250.1)	846.4545	3	APLGTGVVAAVDVEDTPLGTTGEVLR		-	-	-	-	-		-		-		
	683.1170	4	VTTEETRPGIISSIFNTVTGTLGHAK		-	-	-	-	-		-		-		
	643.2995	2	DATVNTTSEYGK		-	-	-	-	-		-		-		
late embryogenesis abundant protein Dc3-like (OTG27147.1)	575.2833	2	TGGIMQQTGEK		+	+	-	+	+		-		-		
	700.8139	2	ESADQTGSYVSEK		-	-	-	+	+		-		-		
	483.2324	2	TGQMMGNVK		-	-	-	-	-		-		-		
isocitrate lyase (OTG28376.1)	844.9412	2	TDAVGATLIQTNIDTR		-	-	-	-	-		-		-		
	1027.4912	2	TFSDAVVDAIGAMNVSEAEK		-	-	-	-	-		-		-		
glutathione peroxidase, partial (ABG35966.1)	884.9393	2	EGEPVVIAPSVSNDEAR		-	-	-	+	-		-		-		
	522.7583	2	DASGQNLPSR		-	-	-	+	+		-		-		
jacalin-related lectin 34-like (XP_022000240.1)	593.0370	4	GTGTGTFGTGGHEGLGTNIGHVEGR		+	+	-	+	+		+		-		
	629.0341	4	HEEHETFVQSDTGGQYQHEVR		-	-	-	-	-		-		-		
	810.8574	2	GHVDSGTGMEYTPGGR		-	-	-	-	-		-		-		
	792.8583	2	TGGIMGEYGSTGQGNR		-	-	-	+	-		-		-		
	897.7539	3	SIGQTGSQGLGTEPGSHGGIMGDYGATR		-	-	-	+	-		-		-		
	422.8753	3	QTDEHGNPIQK		-	-	-	+	-		-		-		
cold shock protein CS66-like (XP_022012178.1)	684.6445	3	DVYGGTGIGTGGHGHGDTHEK		-	-	-	-	-		-		-		
	625.3018	4	STGQTGYQGLGTDAGHHQGLGQATR		-	-	-	+	+		-		-		
	588.6166	3	IIHGTGGTGTDYNTSGGR		-	+	-	+	+		-		-		
	588.7863	2	QVDEYGNPVR		-	-	-	-	-		-		-		

	814.3823	2	TGTGSYGTGQDQGLGTK		-	-	-	-	-		-		-		
	1047.4715	2	LPGGHSTDEQTTTTGYGDTR		-	-	-	-	-		-		-		
peroxygenase-like (XP_022040985.1)	843.0526	3	VMAMADGNALTPEADCQPLTHER		-	-	-	+	-		-		-		
	655.3340	2	VAFDPFGWTAAK		-	-	-	+	+		-		-		
late embryogenesis abundant protein D-34-like (OTG22301.1; XP_021969614.1)	836.9370	2	LGDVLTDASSLLPEDK		-	-	-	+	+		-		-		
	545.2770	2	MEVMPGGVAAK		-	-	-	+	+		-		-		
	818.0596	3	AITPEDAATMQSAENMVLGHTHK		-	-	-	-	-		-		-		
	763.8787	2	YGDVFQVSGELANK		-	-	-	-	-		-		-		
	1032.4861	2	NQPEMATYPGGVAASMAAAAR		-	-	-	-	-		-		-		
chaperone protein ClpB1-like (OTG16327.1)	593.2971	2	GDVPNNLSDVR		-	-	-	-	-		-		-		
putative aspartic proteinase A1 (OTG31235.1)/ cyprosin-like (XP_022028305.1)	742.3492	3	HAGEEEGGELVFGGVDPNHFK		-	-	-	+	-		-		-		
	841.4453	2	FDGILGLGYQDISVGK		-	-	-	+	+		-		-		
	979.1399	3	IPSPMGESAVDCQTLSNMPNIAFTIGGK		-	-	-	+	-		-		-		
putative epoxide hydrolase (OTF98057.1)	595.3464	2	INDIVLDFIK		-	-	-	+	-		-		-		
	958.8392	3	NDVPLLQQIVVLEGAGHFLSQENPEK		-	-	-	+	-		-		-		
	798.3846	3	FQEPGVMEAEIESYGTEHVLK		-	-	-	+	-		-		-		
vicilin-like seed storage protein At2g18540 (OTG22315.1|XP_021969630.1)	988.0245	2	VLQSTFSVPEEVIEELR		-	-	-	+	-		-		-		
	663.6701	3	GGENQPLIVDGQFEISASK		-	-	-	+	-		-		-		
	718.2871	2	GGCGGGWQEEEGGR		-	-	-	-	-		-		-		
	560.5978	3	WPLMSSECGEVSAVK		-	-	-	-	-		-		-		
oleosin 16.4 kDa-like (OTF98883.1|XP_022005568.1)	600.9731	3	AHDIGPEGAVHAGSAVGGAK		-	+	+	+	+		+		-		
	431.8961	3	LQDLVEYTGQK		-	+	+	+	+		+		-		
	491.5927	3	QITGTVPEQVDSAK		-	+	+	+	+		+		-		
	427.9725	4	HPQQVQVHTVHDQR		-	-	+	+	+		-		-		
11-beta-hydroxysteroid dehydrogenase-like 5 (OTG27680.1|XP_022034099.1)	739.3640	2	EVQASGSPVEEFAK		-	-	-	-	-		-		-		
11S globulin seed storage protein G3-like (XP_021988017.1)	973.9836	2	VQIVDNQGNSVFDNELR		-	+	+	+	+		+		-		
putative 11-S seed storage protein	388.1664	3	GMDSSADSHQK		-	-	-	+	+		+		-		
	885.4558	3	NQEVVAIIVDDVNNPANQLDFQAK		-	+	+	+	+		+		-		

(OTG28570.1)	610.8044	2	SPFGGQEELTR		+	+	+	+	+		+		-		
2S seed storage albumin 1 (XP_021993221.1)	655.7945	2	GQFGGQEMETAR		+	+	+	+	+		+		-		
vicilin-like seed storage protein At2g28490 (XP_022001204.1)	1050.9530	2	NDYGWSVEVDGDDYEPLK		-	-	-	+	-		-		-		
oleosin, partial (CAA44224.1)	669.3211	2	GTLQDAGEYAGQK		-	+	+	+	+		+		-		
	747.3840	2	QTAGSVPESLDYVK		-	-	+	+	+		+		-		
oleosin (CAA55348.1)	684.3465	2	HHVTTTQPQYR		-	-	-	+	+		+		-		
	619.3081	2	LQDVGEYTGQK		-	+	+	+	+		+		-		
